# External validation of predictive models of sexual, urinary, bowel and hormonal function after surgery in prostate cancer subjects

**DOI:** 10.1186/s12894-023-01373-9

**Published:** 2024-01-02

**Authors:** Matthew A. Borg, Michael E. O’Callaghan, Kim L. Moretti, Andrew D. Vincent

**Affiliations:** 1 School of Public Health, University of Adelaide, Adelaide, SA Australia; 2 Urology Unit, Flinders Medical Centre, Bedford Park, SA Australia; 3 South Australian Prostate Cancer Clinical Outcomes Collaborative, Adelaide, SA Australia; 4 Flinders Health and Medical Research Institute, Flinders University, Bedford Park, SA Australia; 5 Discipline of Medicine, The University of Adelaide, Adelaide, SA Australia; 6 Discipline of Surgery, The University of Adelaide, Adelaide, SA Australia; 7 Cancer Epidemiology and Population Health Allied Health & Human Performance, University of South Australia, Adelaide, SA Australia; 8 Faculty of Medicine Nursing and Health Sciences, School of Public Health and Preventative Medicine Monash University, Melbourne, Victoria Australia; 9 Freemasons Centre for Male Health & Wellbeing, University of Adelaide, Adelaide, SA Australia

**Keywords:** Prostate cancer, Prostatectomy, Patient-reported outcomes, External validation

## Abstract

**Background:**

In 2020, a research group published five linear longitudinal models, predict Expanded Prostate Cancer Index Composite-26 (EPIC-26) scores post-treatment for radical prostatectomy, external beam radiotherapy and active surveillance collectively in US patients with localized prostate cancer.

**Methods:**

Our study externally validates the five prediction models for patient reported outcomes post-surgery for localised prostate cancer. The models’ calibration, fit, variance explained and discrimination (concordance-indices) were assessed. Two Australian validation cohorts 1 and 2 years post-prostatectomy were constructed, consisting of 669 and 439 subjects, respectively (750 in total). Patient reported function in five domains post-prostatectomy: sexual, bowel, hormonal, urinary incontinence and other urinary dysfunction (irritation/obstruction). Domain function was assessed using the EPIC-26 questionnaire.

**Results:**

1 year post-surgery, R^2^ was highest for the sexual domain (35%, SD = 0.02), lower for the bowel (21%, SD = 0.03) and hormone (15%, SD = 0.03) domains, and close to zero for urinary incontinence (1%, SD = 0.01) and irritation/obstruction (− 5%, SD = 0.04). Calibration slopes for these five models were 1.04 (SD = 0.04), 0.84 (SD = 0.06), 0.85 (SD = 0.06), 1.16 (SD = 0.13) and 0.45 (SD = 0.04), respectively. Calibration-in-the-large values were − 2.2 (SD = 0.6), 2.1 (SD = 0.01), 5.1 (SD = 0.1), 9.6 (SD = 0.9) and 4.0 (SD = 0.2), respectively. Concordance-indices were 0.73, 0.70, 0.70, 0.58 and 0.62, respectively (all had SD = 0.01). Mean absolute error and root mean square error were similar across the validation and development cohorts. The validation measures were largely similar at 2 years post-surgery.

**Conclusions:**

The sexual, bowel and hormone domain models validated well and show promise for accurately predicting patient reported outcomes in a non-US surgical population. The urinary domain models validated poorly and may require recalibration or revision.

**Supplementary Information:**

The online version contains supplementary material available at 10.1186/s12894-023-01373-9.

## Introduction

Prostate cancer is the most common cancer globally in males with an estimated 16,741 new cases in Australia in 2020 [1, 2]. Prostatectomy is a standard of care treatment for localized prostate cancer [3]. However, prostate cancer often progresses slowly without noticeable symptoms [2]; patients with localized prostate cancer commonly die from other causes [4]. This complicates the rationale for treating prostate cancer. Treatment choices should incorporate both patient and clinical factors and avoid overtreatment of prostate cancer [4]. Prostatectomy and its associated adverse events on quality of life, including erectile dysfunction and urinary incontinence, may outweigh the benefit of treatment [4]. Hence patient reported outcomes measures (PROMs) may be equally as important as clinical outcomes such as survival. PROMs can provide a more holistic interpretation of treatment benefit and improve clinical decision making, patient-doctor communication, and patient outcomes [5–7].

Formal prediction models can assist clinicians in accurately predicting PROMs post-prostatectomy to determine the potential treatment benefit of prostatectomy [8]. Laviana et al. recently developed five publicly available online models to predict PROMs after three potential treatment options in pre-treatment US patients with clinical localized stage T1-T2 prostate cancer [9, 10]. The treatment options were radical prostatectomy (RP), external beam radiation therapy (EBRT) and active surveillance (AS); they are analysed collectively. The models reported good calibration and model fit [9]. External validation is important to determine if the model is useful for predicting outcomes in populations similar to, but different from, the development population and if they can be applied individually to each specific treatment [11]. We were also concerned that one treatment (RP) is historically associated with higher rates of sexual dysfunction and incontinence, and another (AS) has potentially very little incidence [12]. Given that the three therapies were analysed collectively and not individually, this study externally validates the online tool and determines its utility at 1- and 2-years post-surgery.

## Materials/subjects and methods

### Development models

Laviana et al. developed five longitudinal linear regression models post-treatment for localized prostate cancer [9]. Each model estimated one EPIC-26 domain score: sexual function, urinary incontinence, urinary irritation/obstruction, bowel function or hormonal function. Scores ranged from 0 to 100 where higher numbers indicate better outcomes [13]. Laviana et al. excluded the last sexual domain question (utilizing five questions instead of six); we validate the 5-question sexual domain model in the main analysis and the 6-question model as a supplementary analysis. Predictor variables were time post-treatment, primary treatment choice (RP, AS, and EBRT), age at diagnosis, race, baseline domain score, overall health, prostate specific antigen (PSA) and Gleason score. Overall health was assessed with the SF-36 Health Survey question “In general, would you say your health is Poor, Fair, Good, Very good or Excellent [14].” The exclusion criteria were receiving androgen deprivation therapy, brachytherapy or cryoablation, age > 80, PSA ≥ 50 ng/dL and non-localized prostate cancer. Laviana et al. analysed all three primary treatments choices collectively in the same models.

### Validation cohort

The South Australian Prostate Cancer Clinical Outcomes Collaborative (SA-PCCOC) database is a population-based registry currently recruiting over 90% of newly diagnosed prostate cancer cases in the state of South Australia, including over 19,000 men [15]. Established in 1998, the registry collects data from collaborating public and private institutions and clinicians [15, 16]. PROMs are collected before treatment commences and at 3, 6, 12, 24 and 60 months post-primary treatment. Participants were mailed paper copies of the Expanded Prostate Cancer Index Composite-26 (EPIC-26) questionnaires and a reply-paid envelope for response.

We sought to verify the Laviana et al. models specifically for RP and followed their inclusion criteria in accordance with the transparent reporting of a multivariable prediction model for individual prognosis or diagnosis (TRIPOD) checklist (Supplementary Table 1) [17]. De-identified data was obtained from SA-PCCOC for post-prostatectomy patients diagnosed between January 2007 and March 2020. The registry does not record participant race; hence we assumed all subjects were Caucasian, which likely matched both the majority of SA-PCCOC patients and 74% of subjects in Laviana’s development cohort [9]. Patients were included only if they completed both a pre- and post-surgery EPIC-26 questionnaire. Post-surgery assessments were required to be within 8–16 or 20–28 months post-prostatectomy, defining a 1- and a 2-year validation cohort, respectively. If a patient completed multiple assessments per time range, only the closest to the target time was included. The 1- and 2-year survey time points are those where SA-PCCOC has the largest quantity of complete data.

SA-PCCOC data are not publicly available but can be obtained after establishing a Data Use Agreement with SA-PCCOC, which has permission to authorize data accessibility from the Southern Australian Clinical Human Research Ethics Committee (SALHN HREC). The data collected by SA-PCCOC has been approved by the SALHN HREC (protocol 307.14).

### Statistical methods

Model predictive values were assessed for calibration – the agreement between the observed endpoints and predictions (calibration-in-the-large (CL) and calibration slope (CS)) – predictive accuracy (mean absolute error (MAE) and root mean square error (RMSE)), proportion of variance explained (R^2^) and discrimination – the effectiveness at predicting which scores would be better or worse (concordance-index, abbreviated as c-index) [11, 18]. CL was estimated as the intercept in a regression with the predicted EPIC-26 score as an offset, and CS was the estimated slope of the predicted score in a univariable regression. Theoretically perfect CL and CS values are 0 and 1, respectively. Because a large proportion of the EPIC-26 domain scores were close to either the upper (100) or lower (0) boundaries, the two calibration metrics were calculated using quantile (median) regression, with linear (mean) regression reported as a supplementary analysis. Predicted versus observed EPIC-26 scores are shown graphically locally weighted smoothing regressions superimposed over scatter plots. R^2^ is calculated as one minus the ratio of residual and total variance i.e. $1-\frac{\sum_{\textrm{i}=1}^{\textrm{n}}{\left({\textrm{y}}_{\textrm{i}}-{\hat{\textrm{y}}}_{\textrm{i}}\right)}^2.}{\sum_{\textrm{i}=1}^{\textrm{n}}{\left({\textrm{y}}_{\textrm{i}}-\overline{\textrm{y}}\right)}^2}$ [18]. For predictive models, R^2^ can be negative [18]. MAE and RMSE are defined as MAE = $\frac{\sum_{\textrm{i}=1}^{\textrm{n}}\left|{\hat{\textrm{y}}}_{\textrm{i}}-{\textrm{y}}_{\textrm{i}}\right|}{\textrm{n}}$ and RMSE = $\sqrt{\frac{\sum_{\textrm{i}=1}^{\textrm{n}}{\left({\hat{\textrm{y}}}_{\textrm{i}}-{\textrm{y}}_{\textrm{i}}\right)}^2}{\textrm{n}}}$. The c-index is equal to the probability that, for two randomly selected subjects after surgery, the subject with a higher predicted score has a higher observed score instead of a lower score i.e. $\text{P}\left(\left({\widehat{\text{y}}}_\text{i}-{\widehat{\text{y}}}_\text{j}\right)\left({\text{y}}_\text{i}-{\text{y}}_\text{j}\right)>0\vert{\widehat{\text{y}}}_\text{i}\neq{\widehat{\text{y}}}_\text{j}\right)$ [19]. CL and discrimination were reported for the first time in these prediction models. The registry database had several patients with unreported pre-surgery PSA or general health assessment; hence multiple imputation using chained equations was performed (100 datasets generated with 100 iterations each) [20]. Missing EPIC-26 domain scores were not imputed. For each validation statistic, means (standard deviations) and median (interquartile ranges) are reported across the 100 imputed datasets. Analyses were performed in R version 4.0.5 using the packages *mice* and *quantreg* [21–23].

## Results

### Validation cohorts

**Fig. 1:**
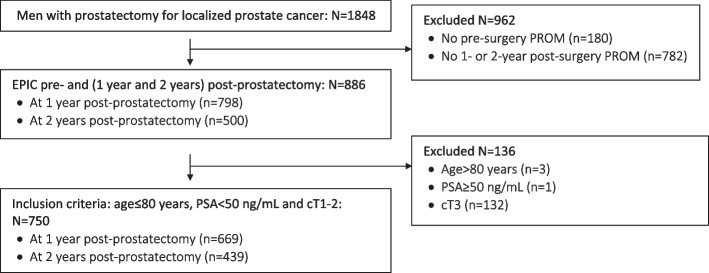
Flow diagram presenting inclusion/exclusion criteria Abbreviations: cT: Clinical T stage, PSA: prostate-specific antigen

Between January 2007 and March 2020, the registry recorded 1848 patients having prostatectomy for localized prostate cancer, 886 of which competed both a pre- and post-surgery EPIC-26 questionnaire. 750 subjects satisfied the inclusion criteria, including 669 and 439 subjects at 1- and 2-years post-surgery, respectively (Fig. 1). Compared with the target paper’s development cohort, our study’s two validation cohorts had slightly higher, but generally similar, age and PSA values and Gleason scores (Table 1). 52% of the development cohort had a Gleason score ≤ 6, but half of the 1-year and 2-year validation cohorts had a score of 3 + 4 (49 and 50%, respectively) and these two cohorts had at least twice as large a proportion of subjects with Gleason scores of 4 + 3 or ≥ 8 compared to the development cohort.

**Table 1:** Summary statistics for patient age and disease characteristics in the development and two validation cohorts

	Laviana 2020	1-year post	2-year post
	*N* = 1402	*N* = 669	*N* = 439
Age (years)			
Mean (SD)		65.4 (6.3)	65.1 (6.2)
Median (range)	62 (57, 66)	66 (41, 78)	66 (41, 78)
Missing	0% (0)	2% (15)	2% (8)
Gleason score			
≤6	52% (723)	12% (82)	10% (44)
3 + 4	30% (413)	49% (329)	50% (220)
4 + 3	10% (145)	22% (150)	21% (93)
≥8	8% (117)	16% (105)	18% (81)
Missing	< 1% (4)	< 1% (3)	< 1% (1)
Prostate specific antigen (ng/dL)			
0- < 4	23% (319)	12% (77)	11% (49)
4- < 10	67% (933)	53% (353)	51% (222)
10- < 20	8% (114)	14% (94)	17% (73)
≥20	3% (36)	2% (13)	3% (11)
Missing	0% (0)	20% (132)	19% (84)
Pathological T stage			
1–2		37% (245)	35% (155)
3–4		50% (332)	54% (237)
Missing		14% (92)	11% (47)
Pathological N and M stage			
N0, M0		40% (269)	43% (187)
N1, M1		3% (19)	3% (13)]
Missing		57% (381)	54% (239)
General health			
Fair		< 1% (5)	1% (5)
Good		19% (128)	11% (49)
Very good		21% (142)	16% (71)
Excellent		6% (39)	5% (24)
Missing		53% (355)	66% (290)

Demographics for the development cohort from Laviana et al. and the validation cohorts (1 year and 2 years post-surgery) from this study, from SA-PCCOC. Only subjects treated with prostatectomy alone are included

Table 2 summarizes the validation cohort EPIC-26 domain scores pre- and post-surgery. The mean pre-surgery sexual scores ranged from 61.2 to 63 and were considerably higher than their post-surgery equivalents (ranging from 29.1 to 37.1). The 1-year and 2-year pre-surgery average urinary incontinence scores of 93.3 and 93.1, respectively, were also higher than the post-surgery scores of 74.6 and 76.1, respectively. However, the 1-year and 2-year pre-surgery mean urinary irritation/obstruction scores of 86.7 and 85.8, respectively were slightly lower than the post-surgery scores of 92.4 and 93.3, respectively. The pre-surgery and post-surgery scores were similar across the bowel and hormone domains. The scores across the 1-year and 2-year cohorts were very similar; the largest difference was a post-surgery sexual score of 34 in the 1-year cohort compared to 29.1 in the 2-year cohort.

**Table 2:** Pre- and post-surgery mean EPIC-26 scores of the two validation cohorts

	1-year post-surgery cohort (N = 669)		2-year post-surgery cohort (N = 439)	
Scores	Pre-surgery	Post-surgery	Pre-surgery	Post-surgery
Sexual (5-question)				
Mean (SD)	61.2 (31.8)	34 (33.2)	61.6 (31.9)	29.1 (29.7)
Median (range)	69.2 (0, 100)	25 (0, 100)	70 (0, 100)	16.7 (0, 100)
Missing	7% (49)	15% (4503)	6% [27]	17% (111)
Sexual (6-question)				
Mean (SD)	62.9 (30)	37.1 (30.6)	63 (30)	31.7 (27.6)
Median (range)	70.8 (0, 100)	26.4 (0, 100)	70.8 (0, 100)	20.8 (0, 100)
Missing	9% (62)	17% (5203)	8% (33)	18% (123)
Urinary incontinence				
Mean (SD)	93.3 (12.7)	74.6 (24.5)	93.1 (13.1)	76.1 (23.8)
Median (Range)	100 (6.2, 100)	79.2 (8.3, 100)	100 (6.2, 100)	79.2 (0, 100)
Missing	3% [23]	10% (2931)	4% [16]	4% [26]
Urinary irritation/obstruction				
Mean (SD)	86.7 (14.9)	92.4 (11.4)	85.8 (15.5)	93.3 (9.5)
Median (Range)	93.8 (18.8, 100)	93.8 (18.8, 100)	93.8 (25, 100)	93.8 (6.2, 100)
Missing	3% [20]	3% (915)	2% [10]	3% [20]
Bowel				
Mean (SD)	94.1 (10.2)	93.2 (11.5)	93.4 (11.5)	93.9 (10.8)
Median (Range)	100 (29.2, 100)	100 (25, 100)	100 (20.8, 100)	100 (25, 100)
Missing	2% [14]	2% (593)	2% [10]	1% [9]
Hormone				
Mean (SD)	94.5 (8.3)	92.4 (12.8)	94.2 (8.8)	93.1 (10.4)
Median (Range)	100 (55, 100)	100 (0, 100)	100 (55, 100)	100 (10, 100)
Missing	5% (35)	8% (2499)	5% [21]	6% (41)

### PROMs – sexual function

**Fig. 2:**
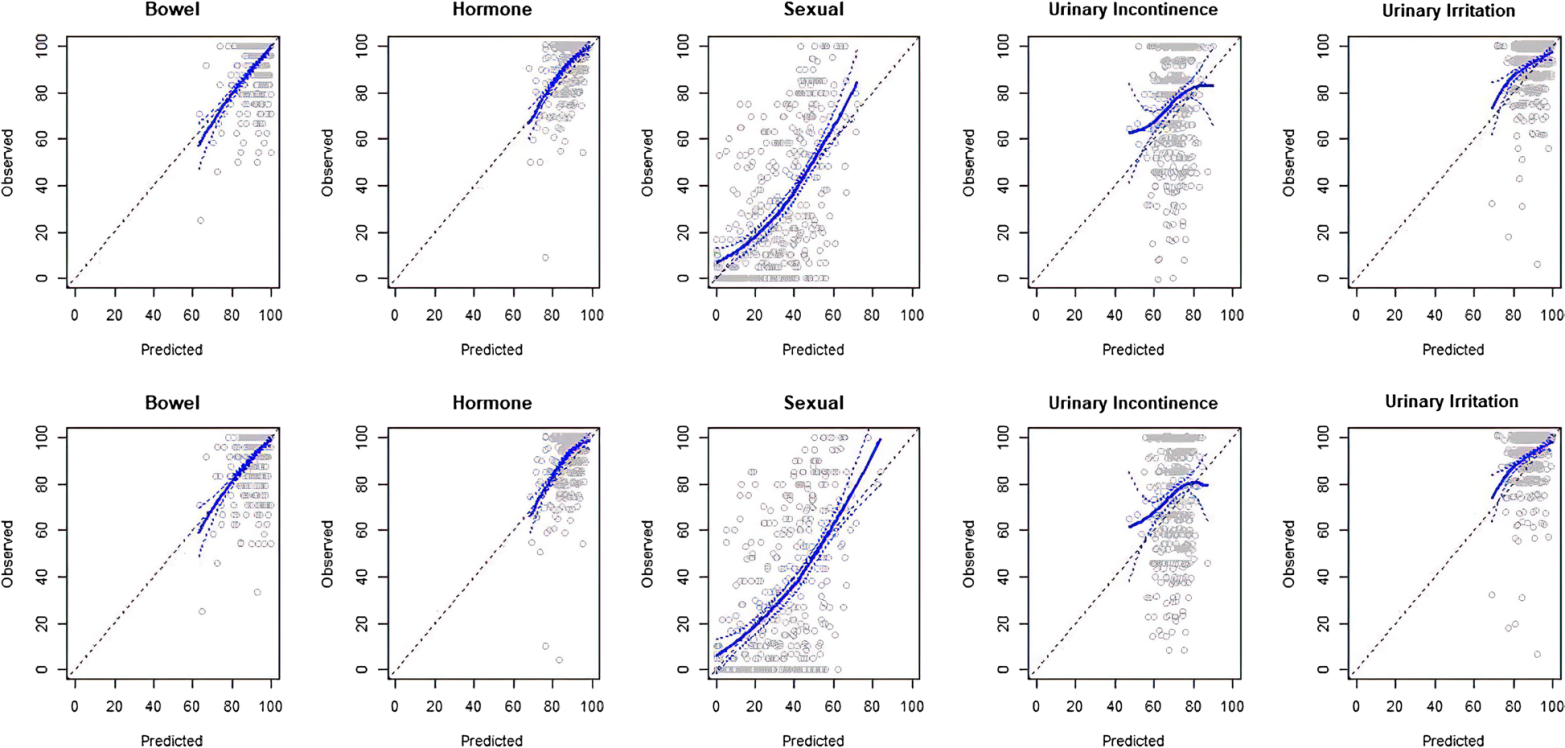
Predicted vs observed EPIC-26 scores Predicted vs observed EPIC-26 scores at 1 year (top row) and 2 years (bottom row) post-prostatectomy for the first imputed set. The blue solid line is a locally weighted smoothing fit with 95% confidence intervals (blue dashed lines), the black diagonal dashed line is the line of perfect prediction

Of the five models, the sexual domain models exhibited the most accurate predictions (Table 3 and Fig. 2). At 1- and 2-years post prostatectomy, the 5-question sexual domain models had the highest variance explained (R^2^ = 0.35, SD = 0.02, respectively) and discrimination (c-index = 0.73 and 0.72, SD = 0.01 and 0.01, respectively), small bias (CL = − 2.2 and − 2.3, SD = 0.6 and 0.7 respectively), and calibration slopes close to one (CS = 1.04 and 1.08, SD = 0.04 and 0.03 respectively). The magnitude of differences between predictions and observations were large (MAE = 19.6 and 20.4, SD = 0.4 and 0.4; RMSE = 24.4 and 25.5, SD = 0.4 and 0.4, respectively) compared to other models. However, this was because the observations were further away from the minimum (0) and maximum (100) score values.

**Table 3:** Mean (standard deviation) of the validation statistics from 100 imputed datasets for 1- and 2-year post-prostatectomy score predictions

	Year	R^2^	CL	CS	MAE	RMSE	c-index
Bowel	1	0.21 (0.03)	2.1 (0.1)	0.84 (0.06)	6.0 (0.2)	9.7 (0.4)	0.70 (0.01)
	2	0.19 (0.03)	3.0 (0.1)	0.75 (0.05)	6.5 (0.2)	9.9 (0.4)	0.71 (0.01)
Hormone	1	0.15 (0.03)	5.1 (0.1)	0.85 (0.06)	7.3 (0.1)	9.3 (0.3)	0.70 (0.01)
	2	0.11 (0.03)	5.4 (0.1)	0.88 (0.06)	8.0 (0.2)	10.9 (0.6)	0.69 (0.01)
Sexual	1	0.35 (0.02)	-2.2 (0.6)	1.04 (0.04)	19.6 (0.4)	24.4 (0.4)	0.73 (0.01)
	2	0.35 (0.02)	−2.3 (0.7)	1.08 (0.03)	20.4 (0.4)	25.5 (0.4)	0.72 (0.01)
Urinary	1	0.01 (0.01)	9.6 (0.9)	1.16 (0.13)	20.1 (0.3)	23.8 (0.3)	0.58 (0.01)
incontinence	2	0.00 (0.01)	10.1 (0.7)	0.99 (0.17)	20.4 (0.3)	23.9 (0.3)	0.57 (0.01)
Urinary irritation/	1	−0.05 (0.04)	4.0 (0.2)	0.45 (0.04)	7.8 (0.2)	10.6 (0.4)	0.62 (0.01)
obstruction	2	−0.06 (0.04)	5.0 (0.2)	0.49 (0.03)	8.2 (0.2)	11.2 (0.3)	0.65 (0.01)

*Abbreviations*: *C-index* Concordance index, *CL* Calibration-in-the-large, *CS* Calibration slope, *MAE* Mean absolute error, *RMSE* Root mean squared error, *SD* Standard deviation

### PROMs – incontinence

The 1- and 2-year urinary incontinence and irritation/obstruction models produced consistently poor validation values. However, the urinary incontinence models had satisfactory calibration slopes (1-year CS = 1.16, SD = 0.13 and 2-year CS = 0.99, SD = 0.17, the latter slope being almost ideal).

### PROMs – bowel and hormonal

Both the bowel and hormone models exhibited reasonable variance explained (1-year bowel and hormone: R^2^ = 0.21 and 0.15, SD = 0.03 and 0.03, respectively), discrimination (c-index = 0.70, SD = 0.01 for both models), bias (CL = 2.1 and 5.1, SD = 0.1 and 0.1, respectively) and calibration slopes (CS = 0.84 and 0.85, SD = 0.06 and 0.06, respectively).

All the 1-year and 2-year values were approximately similar in all domains. The model fit measures (RMSE and MAE) were similar or slightly smaller than the equivalent values in the development cohort, with the exception of the urinary incontinence domain which had slightly larger values. All models exhibited lower R^2^ compared to those in the development cohort. Supplementary Table 2 presents the supplementary results. The 6-question sexual domain model statistics were all similar to the 5-question statistics. The calibration statistics when estimated using linear (mean) regression instead of quantile (median) regression were similar in every domain except for urinary incontinence.

## Discussion

This study externally validates a recently published online tool designed to predict patient symptoms after treatment for localized prostate cancer. The analysis shows excellent predictive performance of the model for sexual function at both 1- and 2-year assessments post-prostatectomy, reasonable performance for bowel and hormonal function, and poor predictive value of urinary incontinence and irritation/obstruction. In particular, these models demonstrated good calibration and discrimination. The online tool would be beneficial for predicting sexual, bowel and hormonal outcomes following prostatectomy. However, the hormonal predictions are not germane to surgery; as seen in Table 2, there is no difference between the three time points. Thus, using the tool to predict post RP hormonal symptoms has little clinical application despite our validation. This can also be concluded for the bowel or rectal function; the descriptive statistics for the bowel domain displayed little change in following prostatectomy. In contrast, there was a large difference in sexual function before and after surgery.

The model performance was worse in the validation cohorts than the development cohort. Although this is to be expected with validation cohorts, the discrepancy could be partially explained by a number of factors. Sexual function is particularly impeded by prostatectomy compared to other domains [12], as shown in the differences between the pre- and post-surgery scores. Our analysis only included surgery, whereas Laviana et al. also included radiotherapy and active surveillance collectively, treatment modalities associated with relatively smaller decreases in sexual scores post-treatment [9]. Surgery has been associated with worse urinary incontinence but improved urinary irritation/obstruction, which matches the validation pre- and post-surgery domain scores, compared to EBRT or AS [12, 24]. This may explain our observed poor correlation for urinary incontinence and irritation/obstruction given we examined RP alone, and the inclusion of AS and EBRT in the development cohort likely resulted in a substantial bias towards the null for surgical subjects. Although the original models considered three different treatments simultaneously, models stratified by treatment may be more specific and meaningful for the relevant therapy. Differences in RP surgical approaches between the two cohorts could also result in differences in PROM outcomes. For example, higher proportions of nerve-sparing surgeries may be associated with improved erectile function and urinary continence [4, 25]. However, the inclusion of AS and ERBT in the development cohort but not the validation cohort is likely to predispose to larger differences between the cohorts than the choice of RP surgical technique.

Differences in model performance can also result from underlying population differences. Although the development and validation cohorts’ summary statistics appear reasonably similar, the validation cohorts had on average higher Gleason scores and, to a small extent, age and PSA. These are all risk factors associated with worse outcomes [26]. RP is more likely to be offered to higher risk patients compared to AS [27], and in particular, AS patients generally have Gleason scores ≤6 [28]. Hence the validation cohorts, which only include subjects who had RP, were likely to have overall stronger risk factors compared to a cohort that includes subjects who elected for AS. Model performance can also vary because of differences in the US and South Australian healthcare systems and socioeconomic factors which have not be addressed in these analyses. Underlying population differences were likely given the lower R^2^ values in the validation cohorts. However, the RMSEs and MAEs were similar between the cohorts. These two statistics are less volatile to population differences and indicate a similar model fit with both data sets despite their differences [29]. This also suggests that the original models were unlikely to be overfitted to the development population.

Our study’s strengths include a large validation cohort size collected across 13 years. The registry has data on most newly diagnosed prostate cancers in South Australia [15], reflecting a high population level recruitment inherent in disease-specific registries. By validating the development models in a surgery-only cohort, this study’s results potentially better reflect the prevalence of PROMs in surgical patients, particularly for sexual and urinary symptoms. Multiple performance statistics were considered for a comprehensive model validation exploring calibration, model fit, variance explained, and discrimination.

SA-PCCOC does not record racial group, and both PSA and general health had substantial missingness, which are the most pronounced study limitations and may obscure underlying population differences between the cohorts. However multiple imputation was implemented to minimise the impact of missing data. As subjects with advanced prostate cancer were excluded, we believe it is unlikely that missing data was greatly impacted by the effect of prostate cancer on subjects’ health. Thus assuming the data was missing at random is reasonable, minimizing potential bias from the multiple imputation. Furthermore, omitting racial group would likely only have a small effect. Racial group in the development models only created a difference in the predicted scores of up to 3.8 in 99% of subjects for the sexual and urinary incontinence domains (races of “White”, “Black”, “Hispanic”, and “Asian”), and even smaller differences no greater than 2.4 for all subjects in the other three domains [9]. These differences are all below the minimum clinically important difference (MCID) for the EPIC-26 instrument [30]. However, our models may have underestimated urinary incontinence scores for the other 1% of subjects (the “Other” racial group), where their predicted score increase in this domain (8.5) exceeded the MCID. The visits assessed in this study differed to those from the original study, adding a 2-year visit but omitting visits occurring at 6 months, 3 years and 5 years. Although caution should be excised for the models’ validity in non-US populations at these timepoints, the generally good performance of these models at 1 and 2 years give evidence to suggest that the validated models will perform well across the full 5-year period analysed in the development study. A study observed similar mean EPIC-26 domain scores at 2- and 3-years in a large cohort of US patients post-prostatectomy almost identical to that of the development cohort [24].

## Conclusions

The examined models perform well for predicting sexual and, to a lesser extent, bowel and hormonal symptoms 1- and 2-years post-surgery in our prostate cancer patients. However, they perform poorly in predicting urinary incontinence and irritation/obstruction. The urinary domain models may benefit from recalibration or revision to better predict PROMs post-prostatectomy in non-US populations. The models for the other three domains are likely suitable for non-US populations and should be considered for implementation into clinical practice.

### Supplementary Information

**Additional file 1: Supplementary Table 1. **TRIPOD Checklist: Prediction Model Validation. **Supplementary Table 2. **Validation statistics for the five prediction models across the 100 imputed datasets of the 1- and 2-year post-prostatectomy cohorts.

## Data Availability

The data used for this study are available from SA-PCCOC. These data cannot be made publicly available as part of the SA-PCCOC Data Use Agreement. A request to receive this data can be made and discussed after contacting SA-PCCOC. SA-PCCOC’s contact details are listed online at https://www.prostatehealth.org.au/contact-us/.

## Abbreviations

AS: Active surveillance
c-index: Concordance-index
CL: Calibration-in-the-large
CS: Calibration slope
EBRT: External beam radiation therapy
EPIC-26: Expanded Prostate Cancer Index Composite-26
MAE: Mean absolute error
PROMs: Patient reported outcomes measures
PSA: Prostate specific antigen
RMSE: Root mean square error
R^2^: Proportion of variance explained
RP: Radical prostatectomy
SA-PCCOC: South Australian Prostate Cancer Clinical Outcomes Collaborative
SD: Standard deviation

## References

[1] Cancer Australia. Prostate cancer in Australia statistics. Canberra, Australia: Australian Government 2021 Available from: https://www.canceraustralia.gov.au/affected-cancer/cancer-types/prostate-cancer/statistics

[2] Leslie SW, Soon-Sutton TL, IA R, Sajjad H, Siref LE, Prostate Cancer (2023). StatPearls.

[3] Sebesta EM, Anderson CB (2017). The surgical Management of Prostate Cancer. Semin Oncol..

[4] Mottet N, Cornford P, van den Bergh RCN, Briers E, Eberli D, De Meerleer G, et al. Prostate Cancer Arnhem. Netherlands Euorp Assoc Urol. 2023; Available from: https://uroweb.org/guidelines/prostate-cancer

[5] Weldring T, Smith SMS (2013). Article Commentary: Patient-Reported Outcomes (PROs) and Patient-Reported Outcome Measures (PROMs).

[6] Nelson EC, Eftimovska E, Lind C, Hager A, Wasson JH, Lindblad S (2015). Patient reported outcome measures in practice. BMJ..

[7] Kingsley C, Patel S (2017). Patient-reported outcome measures and patient-reported experience measures. BJA Educ..

[8] Walz J, Gallina A, Perrotte P, Jeldres C, Trinh Q-D, Hutterer GC (2007). Clinicians are poor raters of life-expectancy before radical prostatectomy or definitive radiotherapy for localized prostate cancer. BJU Int..

[9] Laviana AA, Zhao Z, Huang L-C, Koyama T, Conwill R, Hoffman K (2020). Development and internal validation of a web-based tool to predict sexual, urinary, and bowel function longitudinally after radiation therapy, surgery, or observation. Eur Urol..

[10] Laviana AA, Zhao Z, Huang L-C, Koyama T, Conwill R, Hoffman K, et al. A Prediction Model for Domain Scores for Prostate Cancer Patients. Nashville, Tennessee: USA: CEASAR Investigators. Available from: https://statez.shinyapps.io/PCDSPred/

[11] Steyerberg EW, Vergouwe Y (2014). Towards better clinical prediction models: seven steps for development and an ABCD for validation. Eur Heart J..

[12] Sanda MG, Dunn RL, Michalski J, Sandler HM, Northouse L, Hembroff L (2008). Quality of life and satisfaction with outcome among Prostate-Cancer survivors. N Engl J Med..

[13] Sanda MG, Wei JT, Litwin MS. Scoring Instructions for the Expanded Prostate cancer Index Composite Short Form (EPIC-26). The University of Michigan. Available from: https://medicine.umich.edu/sites/default/files/content/downloads/Scoring%20Instructions%20for%20the%20EPIC%2026.pdf

[14] Ware JE, Snow KK, Kosinski M, Gandek B (1993). SF-36 health survey: manual and interpretation guide.

[15] South Australian Prostate Cancer Clinical Outcomes Collaborative. About Us. Adelaide, Australia: [cited 2020 27 November]. Available from: https://www.prostatehealth.org.au/about-us/

[16] South Australian Prostate Cancer Clinical Outcomes Collaborative. List of Our Collaborators. Adelaide, Australia: [cited 2021 23 December]. Available from: https://www.prostatehealth.org.au/collaborators/list-of-our-collaborators/

[17] Collins GS, Reitsma JB, Altman DG, Moons KGM (2015). Transparent reporting of a multivariable prediction model for individual prognosis or diagnosis (TRIPOD): the TRIPOD statement. Ann Intern Med..

[18] Kvalseth TO (1985). Cautionary note about R2. Am Stat..

[19] Harrell FE. Evaluating the yield of medical tests. JAMA: the. J Am Med Assoc. 1982;247(18):2543 10.1001/jama.1982.03320430047030. 7069920

[20] White IR, Royston P, Wood AM (2011). Multiple imputation using chained equations: issues and guidance for practice. Stat Med..

[21] R Core Team (2021). R: a language and environment for statistical computing.

[22] van Buuren S, Groothuis-Oudshoorn K. Multivariate imputation by chained equations in R. J Stat Softw. 2011;45(3) 10.18637/jss.v045.i03.

[23] Koenker R. quantreg: Quantile Regression. 2021. Available from: https://CRAN.R-project.org/package=quantreg.

[24] Barocas DA, Alvarez J, Resnick MJ, Koyama T, Hoffman KE, Tyson MD (2017). Association between radiation therapy, surgery, or observation for localized Prostate Cancer and patient-reported outcomes after 3 years. J Am Med Assoc..

[25] Haga N, Miyazaki T, Tsubouchi K, Okabe Y, Shibayama K, Emoto D (2021). Comprehensive approach for preserving cavernous nerves and erectile function after radical prostatectomy in the era of robotic surgery. Int J Urol..

[26] Srigley JR, Amin M, Boccon-Gibod L, Egevad L, Epstein JI, Humphrey PA (2005). Prognostic and predictive factors in prostate cancer: historical perspectives and recent international consensus initiatives. Scand J Urol Nephrol..

[27] InformedHealth.org. Localized prostate cancer: Low-risk prostate cancer: Active surveillance or treatment? Cologne, Germany: Institute for Quality and Efficiency in Health Care. [updated 12 March 2020; cited 2020 26 December]. Available from: https://www-ncbi-nlm-nih-gov.proxy.library.adelaide.edu.au/books/NBK487255/

[28] Sathianathen NJ, Konety BR, Crook J, Saad F, Lawrentschuk N (2018). Landmarks in prostate cancer. Nat Rev Urol..

[29] Alexander DLJ, Tropsha A, Winkler DA (2015). Beware of R2: simple, unambiguous assessment of the prediction accuracy of QSAR and QSPR models. J Chem Inf Model..

[30] Skolarus TA, Dunn RL, Sanda MG, Chang P, Greenfield TK, Litwin MS (2015). Minimally important difference for the expanded Prostate Cancer index composite short form. Urol..

