# Supplementary material for: External validation of predictive models of sexual, urinary, bowel and hormonal function after surgery in prostate cancer subjects
Source: BMC Urol. 2024 Jan 2;24:2. doi: 10.1186/s12894-023-01373-9 (PMC10763035; doi:10.1186/s12894-023-01373-9)
Supplement: Supplementary file 1 — Additional file 1: Supplementary Table 1. TRIPOD Checklist: Prediction Model Validation. Supplementary Table 2. Validation statistics for the five prediction models across the 100 imputed datasets of the 1- and 2-year post-prostatectomy cohorts. [file 12894_2023_1373_MOESM1_ESM.docx]

## Supplementary Table 1: TRIPOD Checklist: Prediction Model Validation

| Section/Topic | It | Checklist Item | Page |
| --- | --- | --- | --- |
| Title and abstract | | | |
| Title | 1 | Identify the study as developing and/or validating a multivariable prediction model, the target population, and the outcome to be predicted. | Title page |
| Abstract | 2 | Provide a summary of objectives, study design, setting, participants, sample size, predictors, outcome, statistical analysis, results, and conclusions. | 2-3 |
| Introduction | | | |
| Background and objectives | 3a | Explain the medical context (including whether diagnostic or prognostic) and rationale for developing or validating the multivariable prediction model, including references to existing models. | 3-4 |
|  | 3b | Specify the objectives, including whether the study describes the development or validation of the model or both. | 4 |
| Methods | | | |
| Source of data | 4a | Describe the study design or source of data (e.g., randomized trial, cohort, or registry data), separately for the development and validation data sets, if applicable. | 5 |
|  | 4b | Specify the key study dates, including start of accrual; end of accrual; and, if applicable, end of follow-up. | 5 |
| Participants | 5a | Specify key elements of the study setting (e.g., primary care, secondary care, general population) including number and location of centres. | 5 |
|  | 5b | Describe eligibility criteria for participants. | 5-6 |
|  | 5c | Give details of treatments received, if relevant. | 5 |
| Outcome | 6a | Clearly define the outcome that is predicted by the prediction model, including how and when assessed. | 4-5 |
|  | 6b | Report any actions to blind assessment of the outcome to be predicted. | N/A |
| Predictors | 7a | Clearly define all predictors used in developing or validating the multivariable prediction model, including how and when they were measured. | 4 |
|  | 7b | Report any actions to blind assessment of predictors for the outcome and other predictors. | N/A |
| Sample size | 8 | Explain how the study size was arrived at. | 5 |
| Missing data | 9 | Describe how missing data were handled (e.g., complete-case analysis, single imputation, multiple imputation) with details of any imputation method. | 6-7 |
| Statistical analysis methods | 10c | For validation, describe how the predictions were calculated. | 6 |
|  | 10d | Specify all measures used to assess model performance and, if relevant, to compare multiple models. | 6 |
|  | 10e | Describe any model updating (e.g., recalibration) arising from the validation, if done. | N/A |
| Risk groups | 11 | Provide details on how risk groups were created, if done. | N/A |
| Development vs. validation | 12 | For validation, identify any differences from the development data in setting, eligibility criteria, outcome, and predictors. | 5 |
| Results | | | |
| Participants | 13a | Describe the flow of participants through the study, including the number of participants with and without the outcome and, if applicable, a summary of the follow-up time. A diagram may be helpful. | 7, Figure 1 |
|  | 13b | Describe the characteristics of the participants (basic demographics, clinical features, available predictors), including the number of participants with missing data for predictors and outcome. | 7, Table 1 |
|  | 13c | For validation, show a comparison with the development data of the distribution of important variables (demographics, predictors and outcome). | 7-8, Table 2 |
| Model performance | 16 | Report performance measures (with CIs) for the prediction model. | 8-9, Table 3, Figure 2 |
| Model-updating | 17 | If done, report the results from any model updating (i.e., model specification, model performance). | N/A |
| Discussion | | | |
| Limitations | 18 | Discuss any limitations of the study (such as nonrepresentative sample, few events per predictor, missing data). | 12 |
| Interpretation | 19a | For validation, discuss the results with reference to performance in the development data, and any other validation data. | 10-11 |
|  | 19b | Give an overall interpretation of the results, considering objectives, limitations, results from similar studies, and other relevant evidence. | 13 |
| Implications | 20 | Discuss the potential clinical use of the model and implications for future research. | 10-11 |
| Other information | | | |
| Supplementary information | 21 | Provide information about the availability of supplementary resources, such as study protocol, Web calculator, and data sets. | N/A |
| Funding | 22 | Give the source of funding and the role of the funders for the present study. | N/A |

The TRIPOD (transparent reporting of a multivariable prediction model for individual prognosis or diagnosis) checklist reported by Collins et al. 2015. It is a recommended list of items to be reported in a study validating a single multivariable predictive model for prognosis or diagnosis at the individual subject level.

## Supplementary Table 2: Validation statistics for the five prediction models across the 100 imputed datasets of the 1- and 2-year post-prostatectomy cohorts

| **Year** | **Model** |  | **R^2^** | **Calibration-in-the-large** | | **Calibration slope** | | **MAE** | **RMSE** | **C-index** |
| --- | --- | --- | --- | --- | --- | --- | --- | --- | --- | --- |
|  |  |  |  | **Quantile** | **Linear** | **Quantile** | **Linear** |  |  |  |
| **1** | **Bowel** | Mean (SD) | 0.21 (0.03) | 2.1 (0.1) | -0.6 (0.2) | 0.84 (0.06) | 1.02 (0.07) | 6.0 (0.2) | 9.7 (0.4) | 0.70 (0.01) |
|  |  | Median (range) | 0.21 (0.19, 0.24) | 2.1 (2.1, 2.1) | -0.6 (-0.7, -0.4) | 0.84 (0.81, 0.89) | 1.02 (0.97, 1.06) | 6.0 (5.9, 6.2) | 9.8 (9.4, 10.1) | 0.70 (0.69, 0.71) |
|  | **Hormone** | Mean (SD) | 0.15 (0.03) | 5.1 (0.1) | 3.2 (0.2) | 0.85 (0.06) | 0.93 (0.06) | 7.3 (0.1) | 9.3 (0.3) | 0.70 (0.01) |
|  |  | Median (range) | 0.15 (0.12, 0.17) | 5.1 (5.0, 5.2) | 3.2 (3.0, 3.3) | 0.85 (0.81, 0.89) | 0.93 (0.89, 0.98) | 7.3 (7.3, 7.4) | 9.4 (9.2, 9.5) | 0.70 (0.70, 0.71) |
|  | **Sexual (5Q)** | Mean (SD) | 0.35 (0.02) | -2.2 (0.6) | 0.3 (0.5) | 1.04 (0.04) | 1.01 (0.03) | 19.6 (0.4) | 24.4 (0.4) | 0.73 (0.01) |
|  |  | Median (range) | 0.34 (0.33, 0.36) | -2.3 (-2.4, -1.9) | 0.3 (-0.1, 0.7) | 1.04 (1.02, 1.07) | 1.01 (0.99, 1.03) | 19.6 (19.4, 19.9) | 24.4 (24.2, 24.6) | 0.73 (0.72, 0.73) |
|  | **Sexual (6Q)** | Mean (SD) | 0.33 (0.02) | 0.9 (0.9) | 2.7 (0.5) | 0.94 (0.04) | 0.95 (0.03) | 18.3 (0.4) | 23.0 (0.4) | 0.71 (0.01) |
|  |  | Med (range) | 0.33 (0.32, 0.34) | 0.7 (0.2, 1.5) | 2.7 (2.4, 3.0) | 0.95 (0.92, 0.97) | 0.95 (0.94, 0.97) | 18.3 (18.1, 18.5) | 23.0 (22.8, 23.3) | 0.71 (0.70, 0.71) |
|  | **Urinary**  **incontinence** | Mean (SD) | 0.01 (0.01) | 9.6 (0.9) | 5.0 (0.5) | 1.16 (0.13) | 0.86 (0.08) | 20.1 (0.3) | 23.8 (0.3) | 0.58 (0.01) |
|  |  | Median (range) | 0.01 (0.00, 0.02) | 9.6 (8.8, 10.1) | 5.0 (4.7, 5.2) | 1.14 (1.09, 1.25) | 0.86 (0.81, 0.91) | 20.2 (20.0, 20.3) | 23.8 (23.6, 24.0) | 0.58 (0.58, 0.58) |
|  | **Urinary irritation/**  **obstruction** | Mean (SD) | -0.05 (0.04) | 4.0 (0.2) | 2.4 (0.2) | 0.45 (0.04) | 0.52 (0.07) | 7.8 (0.2) | 10.6 (0.4) | 0.62 (0.01) |
|  |  | Median (range) | -0.05 (-0.08, -0.02) | 4.0 (3.8, 4.1) | 2.4 (2.3, 2.5) | 0.45 (0.43, 0.47) | 0.51 (0.48, 0.56) | 7.8 (7.7, 7.8) | 10.6 (10.4, 10.8) | 0.62 (0.61, 0.62) |
| **2** | **Bowel** | Mean (SD) | 0.19 (0.03) | 3.0 (0.1) | 0.3 (0.2) | 0.75 (0.05) | 0.93 (0.06) | 6.5 (0.2) | 9.9 (0.4) | 0.71 (0.01) |
|  |  | Median (range) | 0.19 (0.17, 0.21) | 3.1 (3.0, 3.1) | 0.3 (0.1, 0.4) | 0.74 (0.71, 0.77) | 0.93 (0.88, 0.97) | 6.5 (6.3, 6.6) | 10.0 (9.6, 10.2) | 0.71 (0.70, 0.71) |
|  | **Hormone** | Mean (SD) | 0.11 (0.03) | 5.4 (0.1) | 3.1 (0.3) | 0.88 (0.06) | 0.95 (0.07) | 8.0 (0.2) | 10.9 (0.6) | 0.69 (0.01) |
|  |  | Median (range) | 0.11 (0.10, 0.13) | 5.4 (5.3, 5.5) | 3.1 (3.0, 3.3) | 0.90 (0.83, 0.92) | 0.95 (0.91, 1.00) | 8.0 (7.9, 8.2) | 10.9 (10.5, 11.4) | 0.69 (0.68, 0.70) |
|  | **Sexual (5Q)** | Mean (SD) | 0.35 (0.02) | -2.3 (0.7) | 0.0 (0.5) | 1.08 (0.03) | 1.00 (0.02) | 20.4 (0.4) | 25.5 (0.4) | 0.72 (0.01) |
|  |  | Median (range) | 0.35 (0.34, 0.36) | -2.3 (-2.7, -1.7) | -0.1 (-0.3, 0.2) | 1.08 (1.06, 1.10) | 1.00 (0.98, 1.02) | 20.4 (20.2, 20.7) | 25.6 (25.3, 25.8) | 0.73 (0.72, 0.73) |
|  | **Sexual (6Q)** | Mean (SD) | 0.34 (0.02) | 0.9 (0.9) | 2.6 (0.5) | 1.01 (0.03) | 0.96 (0.02) | 19.0 (0.4) | 24.0 (0.4) | 0.71 (0.01) |
|  |  | Median (range) | 0.34 (0.33, 0.35) | 0.6 (0.0, 1.6) | 2.5 (2.3, 2.8) | 1.01 (0.99, 1.03) | 0.96 (0.94, 0.97) | 19.0 (18.8, 19.3) | 24.0 (23.8, 24.3) | 0.71 (0.70, 0.71) |
|  | **Urinary**  **incontinence** | Mean (SD) | 0.00 (0.01) | 10.1 (0.7) | 4.8 (0.5) | 0.99 (0.17) | 0.80 (0.09) | 20.4 (0.3) | 23.9 (0.3) | 0.57 (0.01) |
|  |  | Median (range) | 0.00 (-0.01, 0.01) | 10.3 (9.6, 10.5) | 4.7 (4.4, 5.0) | 0.97 (0.87, 1.13) | 0.79 (0.74, 0.87) | 20.4 (20.3, 20.6) | 23.9 (23.7, 24.1) | 0.57 (0.56, 0.57) |
|  | **Urinary irritation/**  **obstruction** | Mean (SD) | -0.06 (0.04) | 5.0 (0.2) | 3.2 (0.2) | 0.49 (0.03) | 0.55 (0.06) | 8.2 (0.2) | 11.2 (0.3) | 0.65 (0.01) |
|  |  | Median (range) | -0.06 (-0.09, -0.03) | 5.0 (4.8, 5.1) | 3.2 (3.1, 3.4) | 0.49 (0.46, 0.51) | 0.55 (0.51, 0.59) | 8.2 (8.1, 8.3) | 11.3 (11.1, 11.5) | 0.65 (0.64, 0.65) |

The sexual domain score has been validated both as the 5 question and full 6 question scale. Calibration has been calculated both using quantile (median) and linear (mean) regression. Abbreviations: 5Q: 5-questions, 6Q: 6-questions, C-index: concordance index, MAE: mean absolute error, RMSE: root mean squared error, SD: standard deviation.
